# The Differential Translation Capabilities of the Human *DHFR2* Gene Indicates a Developmental and Tissue-Specific Endogenous Protein of Low Abundance

**DOI:** 10.1016/j.mcpro.2024.100718

**Published:** 2024-01-14

**Authors:** Niamh Bookey, Paola Drago, Kit-Yi Leung, Linda Hughes, Aoife MacCooey, Mari Ozaki, Michael Henry, Sandra C.P. De Castro, Ivan Doykov, Wendy E. Heywood, Kevin Mills, Michelle M. Murphy, Pere Cavallé-Busquets, Susan Campbell, Denise Burtenshaw, Paula Meleady, Paul A. Chill, Nicholas D.E. Greene, Anne Parle-McDermott

**Affiliations:** 1 School of Biotechnology, Dublin City University, Dublin, Ireland; 2 DCU Life Sciences Institute, Dublin City University, Dublin, Ireland; 3 Developmental Biology and Cancer Department, UCL Great Ormond Street Institute of Child Health, University College London, London, UK; 4 Translational Mass Spectrometry Research Group, UCL Great Ormond Street Institute of Child Health, University College London, London, UK; 5 Area of Preventive Medicine and Public Health, Department of Basic Medical Sciences, Faculty of Medicine and Health Sciences, Universitat Rovira i Virgili, IISPV and CIBERobn (Instituto de Salud Carlos III), Reus, Spain; 6 Area of Obstetrics, Hospital Universitari Sant Joan de Reus, IISPV and CIBERobn (Instituto de Salud Carlos III), Reus, Spain; 7 Sheffield Hallam University, Department of Biosciences and Chemistry, Sheffield, UK

**Keywords:** DHFR2, DHFR, translation, dihydrofolate reductase, embryogenesis

## Abstract

A functional role has been ascribed to the human dihydrofolate reductase 2 (*DHFR2*) gene based on the enzymatic activity of recombinant versions of the predicted translated protein. However, the *in vivo* function is still unclear. The high amino acid sequence identity (92%) between DHFR2 and its parental homolog, DHFR, makes analysis of the endogenous protein challenging. This paper describes a targeted mass spectrometry proteomics approach in several human cell lines and tissue types to identify DHFR2-specific peptides as evidence of its translation. We show definitive evidence that the DHFR2 activity in the mitochondria is in fact mediated by DHFR, and not DHFR2. Analysis of Ribo-seq data and an experimental assessment of ribosome association using a sucrose cushion showed that the two main Ensembl annotated mRNA isoforms of DHFR2, 201 and 202, are differentially associated with the ribosome. This indicates a functional role at both the RNA and protein level. However, we were unable to detect DHFR2 protein at a detectable level in most cell types examined despite various RNA isoforms of DHFR2 being relatively abundant. We did detect a DHFR2-specific peptide in embryonic heart, indicating that the protein may have a specific role during embryogenesis. We propose that the main functionality of the *DHFR2* gene in adult cells is likely to arise at the RNA level.

## Highlights

• Dihydrofolate reductase activity of the mitochondria is derived from the DHFR enzyme and not DHFR2.
• The *DHFR2* gene is transcribed but not translated at a detectable level in most cells and tissues.
• *DHFR2* may function at the RNA level.
• DHFR2 protein is detectable at low abundance in human embryonic heart.

## Graphical Abstract

**Figure:**
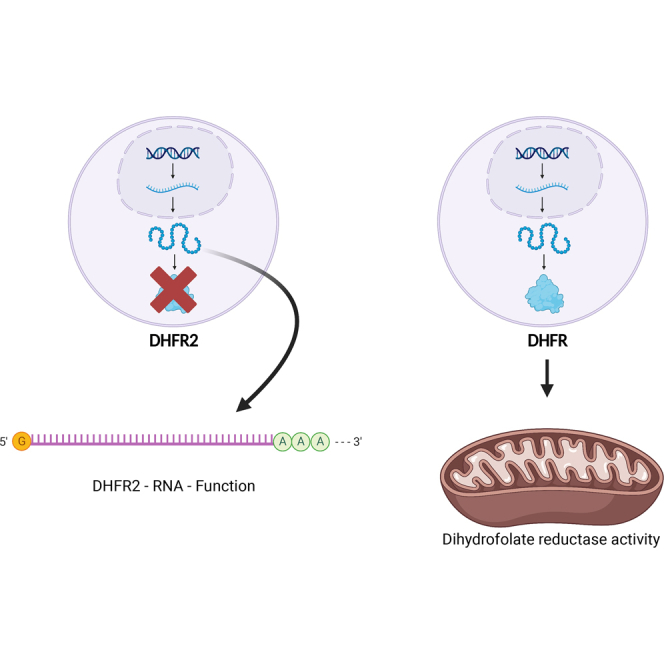

## In Brief

We demonstrate that mitochondrial dihydrofolate reductase activity, detectable in human cells, is derived from *DHFR* and not *DHFR2*. As many antifolate drugs are not transported into the mitochondria, this finding can help enhance drug efficacy. Although *DHFR2* is transcribed, our targeted proteomics analysis did not find evidence of a translated protein in adult cells. We conclude that the main function of the *DHFR2* gene is not to produce a translated enzyme and its functionality may lie at the RNA level.

Dihydrofolate reductase (DHFR) activity is an important enzymatic function that resides within the one carbon metabolism (OCM) pathway (1). This pathway supplies one carbon units for a number of essential cellular processes, including nucleotide synthesis and is therefore particularly active during periods of cellular proliferation, such as embryogenesis (2). Reductase enzyme activity has been found in three separate compartments of mammalian cells where subsets of folate-dependent enzymes are localized, that is, the cytoplasm, nucleus, and mitochondria (3, 4, 5, 6). This enzyme activity is necessary for recycling one carbon units to the biologically active tetrahydrofolate form (cytoplasm) and as an on-site supplier of one carbon units for *de novo* thymidylate synthesis in the nucleus and mitochondria (1, 3, 4, 7). It generates pools of dTMP from dUMP with the help of thymidylate synthase (TYMS) and serine hydroxymethyltransferase (SHMT) (1). It is also the only enzyme that can reduce the synthetic form of folate, folic acid, to tetrahydrofolate to facilitate its entry into the OCM pathway. Its central role in cellular metabolism in support of cell division and proliferation has made DHFR an attractive and successful drug target. One of the classical antifolate drugs, methotrexate, is a direct inhibitor of DHFR, resulting in the cessation of DNA replication as the pools of thymidylate and purines diminish, inevitably resulting in cell death (8). Amplification of the *DHFR* gene is a known mechanism of antifolate drug resistance resulting in extrachromosomal material known as double minutes (9, 10, 11, 12, 13, 14, 15, 16). Its usefulness in the treatment of cancer and other human diseases has meant that the DHFR enzyme has been well studied. However, like many genes, the *DHFR* gene has several highly homologous family members that arose due to historic gene duplication events.

The *DHFR* gene family consists of the originally characterized reductase enzyme, *DHFR*, on chromosome 5, a retrogene known as *DHFR2* on chromosome 3 (formerly known as DHFRL1) and several processed pseudogenes on chromosomes 2, 6, and 18 (17, 18, 19, 20, 21). Our previous research (22) and that of Anderson *et al.* (4) indicated that a recombinant version of DHFR2 localized to the mitochondria and is the reductase activity that drives *de novo* thymidylate synthesis to support mitochondrial DNA replication (4, 22). This was in contrast to our findings in rodents, where only one active reductase is found in all compartments in both mouse and rat (6). However, our previous analysis of human cells involved recombinant versions of DHFR2 and confirmation of the localization and translation of endogenous DHFR2 protein remained to be confirmed. In line with the Human Proteome Project terminology (23); we strove to provide PE1 for DHFR2, that is, protein evidence 1, which requires direct evidence of the natural protein by Mass Spectrometry, Edman sequencing, X-ray NMR, or antibody data. Evidence for natural DHFR2 prior to this study was restricted to PE2, which is evidence of the mRNA transcript only. DHFR2 is currently misannotated as PE1 (https://www.nextprot.org/about/protein-existence). Public databases (as summarised at: https://www.proteinatlas.org/ENSG00000178700-DHFR2/summary/rna) have ample evidence of multiple DHFR2 transcripts across many human cell lines and tissues and as confirmed by our previous publication and that of others (4, 22). It is worth noting, that while the Human Protein Atlas database (https://www.proteinatlas.org/ENSG00000178700-DHFR2/summary/antibody) does indicate immunohistochemistry data that is specific to the DHFR2 protein; it is annotated as “uncertain” and the antibodies used would have crossreacted with DHFR given their high amino acid sequence identity (92%) (22) and the 97% identity of the recombinant protein used to generate the Atlas antibody (HPA051465).

In this paper, we describe a series of proteomic experiments designed to provide PE1 level evidence for the endogenous DHFR2 protein in the context of the relatively abundant DHFR enzyme. We used targeted proteomic analyses on a series of human cell line models (including purified mitochondria) as well as adult and embryonic tissue samples to detect DHFR2-specific peptides. We also assessed the translation capabilities of the two main DHFR2 mRNA isoforms and considered whether DHFR2 protein was subject to ubiquitin-mediated degradation. These analyses demonstrate that the reductase activity of human mitochondria is in fact, the DHFR enzyme (and not DHFR2) in most human cell types. This is paralogous to that of rodents (6) in that, a single enzyme is likely to be responsible for DHFR activity in the majority of cellular compartments. We also show that despite an abundance of different DHFR2 mRNA isoforms across a range of cells and tissues that these are not translated into a detectable protein except in human embryonic heart tissue, indicating a specific requirement for the protein during embryogenesis.

## Experimental Procedures

### Mammalian Cell Culture

The HEK293 cell line (American Type Culture Collection CRL-1573) was grown in Dulbecco’s Modified Eagle’s Medium (DMEM), supplemented with 10% (v/v) fetal bovine serum (FBS), 2 mM L-glutamine, and 1 mM sodium pyruvate. HepG2 cells (Sigma-Aldrich 85011430) were cultured in DMEM hi-glucose, supplemented with 10% (v/v) FBS. IMR32 cells were cultured in Roswell Park Memorial Institute-1640 media supplemented with 10% (v/v) FBS, 1% (w/v) penicillin-streptomycin (P/S), and 2.5% (v/v) Hepes. The undifferentiated human induced pluripotent stem (hiPS) cells (HipSci 77650065) were grown in eight Flex Media and 1% (w/v) P/S solution. The human neuroectodermal progenitor (NEP) stem cells were differentiated from the undifferentiated hiPS cells, according to (24). Human hiPS-derived hepatocytes were differentiated from a human undifferentiated hiPS cell line (101B) according to (25). The HuH7 cell line was grown in DMEM, high glucose, pyruvate, supplemented with 10% (v/v) FBS, 1% (v/v) glutamax, and 1% (w/v) P/S. All cells were grown to approximately 80% confluency in a 5% CO_2_ incubator at 37 °C. The cells were subsequently harvested using trypsin-EDTA (0.25% (v/v)) and stored in a −80 °C freezer.

### Western Blotting and DHFR Enzyme Activity Assay on HEK293 Mitochondrial Fractions

Mitochondrial and cytosolic proteins were extracted from four T75 flasks of HEK293 cells using the Qiagen Qproteome Mitochondria Isolation Kit (37612). The protein concentration was quantified using a Bradford assay (Sigma-Aldrich B6916). Western blotting analysis was performed on the mitochondrial and cytosolic fractions to confirm purity of the fractions. A total of 15 μg of protein was denatured for each fraction at 95 °C for 10 min in NuPage LDS sample buffer (Thermo Fisher Scientific NP0008) and NuPAGE sample reducing agent (Thermo Fisher Scientific NP0004). The samples were run on a 10% SDS-PAGE gel and the proteins were transferred onto a polyvinylidene fluoride membrane using the Pierce G2 Fast Blotter System (Thermo Fisher Scientific). The membrane was blocked in 5% (w/v) nonfat milk diluted in 0.05% (v/v) Tris-buffered saline Tween-20 (TBST) buffer (0.15 M sodium chloride, 0.01 M trizma base, 0.05% (v/v) Tween-20, pH 7.4) for 2 h. The blot was incubated with a mAb GAPDH (Sigma-Aldrich G8795) at a dilution of 1:1000 overnight at 4 °C. The membrane was washed in 0.05% (v/v) TBST buffer and probed with an anti-rabbit horseradish peroxidase (HRP)-conjugated secondary antibody for 1 h. After the blot was washed in 0.05% (v/v) TBST buffer, it was visualized using the SuperSignal West Femto Maximum Sensitivity Substrate (Thermo Fisher Scientific 34095) and GeneGnome Bio Imaging System (Syngene 55000). A second Western blot was carried out on the mitochondrial and cytosolic fractions to investigate the presence of a reductase protein in the mitochondria. This was performed using a primary antibody against DHFR (Abcam ab49881) at a dilution of 1:5000, followed by an anti-rabbit HRP-conjugated secondary antibody.

The endogenous reductase activity was subsequently assessed in the mitochondrial fractions using the dihydrofolate assay kit (Sigma-Aldrich CS03040). The assay was performed according to the manufacturer’s protocol. The experiment was carried out at room temperature and at pH 7.5. The reactions contained 0.15 μg of purified recombinant DHFR protein (positive control) or 100 μg of total mitochondrial protein combined with the kit reagents up to a volume of 500 μl in a 1 ml UVette cuvette. A Biochrom Libra S12 UV/Vis spectrophotometer was used to read the absorbance every 15 s for 2.5 min at 340 nm. The decrease in Δ*A*/min was calculated for the DHFR-positive control and the HEK293 mitochondrial protein sample according to the manufacturer’s instructions. In order to calculate the true Δ*A*/min for the mitochondrial fraction, the background activity needed to be subtracted. The Δ*A*/min background noise was calculated by repeating the assay with the addition of 40 nM methotrexate. The specific activity was then calculated according to the manufacturer’s equation.

### Mitochondrial Fraction Purification, Protein Gel Extraction, and Nano LC-MS/MS

Mitochondrial proteins were extracted from a T175 flask of HepG2 cells using the Qiagen Qproteome mitochondria isolation kit. A total of 43 μg of protein from the mitochondrial sample was resolved on a 12% SDS-PAGE gel (Sigma-Aldrich PCG2006) overnight at 4 °C. The gels were stained for 48 h using Coomassie Brilliant Blue G250 (Merck Millipore 1154440025). One large band was cut out of the gel between 18 to 24 kDa using a 15 to 180 kDa protein ladder as a reference. The large band was subsequently divided into three small pieces and shipped to Alphalyse A/S, Denmark in distilled water. Each of the small bands was desalted, reduced, and alkylated using iodoacetamide. The isolated proteins from each gel slice were enzymatically digested using trypsin and the resulting peptides were concentrated by Speed Vac lyophilization. The lyophilized samples were resuspended in 2% (v/v) acetonitrile (ACN)/0.1% (v/v) formic acid for injection onto the LC-MS/MS instrumentation. The samples were separated for 30 min on the Dionex Nano-LC system and tandem mass spectrometry (MS/MS) analysis was carried out on a Bruker Maxis Impact QTOF instrument. The MS/MS spectra were compared against databases downloaded from NCBInr (67337701 protein sequences; https://www.ncbi.nlm.nih.gov/protein) using the MASCOT (version 2.4) search algorithm. The following search parameters were used for peptide identification: peptide mass tolerance of 10 ppm, carbamidomethylation of cysteine was defined as a fixed modification, and methionine oxidation defined as a variable modification.

### Bioinformatic Analysis of Ribo-SDatasets from the RPFdb v2.0 Beta Database

Ribosome profiling data on 23 human cell lines and tissues were downloaded from the RPFdb v2.0 beta database (http://sysbio.gzzoc.com/rpfdb/). The reads per kilobase per million mapped reads (RPKM) values for the *GAPDH*, *POLG*, *MTHFD1L*, *TYMS*, *DHFR*, and *DHFR2* genes were extracted from the 23 samples and assessed. The *GAPDH* and *POLG* genes were used as ubiquitously expressed controls, with *POLG* specific to the mitochondria. Any sample that had an outlier within these genes was excluded from subsequent analysis. The *MTHFD1*, *TYMS*, and *DHFR* genes were employed as ribosome-bound OCM controls.

### Sucrose Cushion Ultracentrifugation RT-qPCR

Three T175 flasks of HepG2 cells were incubated in serum-free DMEM and 100 μg/ml of cycloheximide (CHX) (Sigma-Aldrich C1988) in a 5% CO_2_ incubator at 37 °C for 5 min. The cells were washed twice with ice-cold PBS containing 100 μg/ml CHX and harvested using a cell scraper. Two flasks were merged for the sample and the remaining flask was for the whole-lysate control (WLC). Cells were collected by centrifugation and washed with ice-cold PBS containing 100 μg/ml CHX. Cells were sheared in 250 μl of CSB buffer (300 mM sorbitol, 20 mM Hepes pH 7.5, 1 mM EDTA, 5 mM MgCl_2_, 10 mM KCl, 10% (v/v) glycerol, 100 μg/ml CHX, Halt Protease, and phosphatase inhibitor cocktail (1x)) with glass bead homogenization for eight 20 s vortexing cycles (2500 rpm), each followed by 20 s on ice. An additional 300 μl of CSB buffer was added. Cellular debris was removed by centrifuging the lysate at 2000*g* for 5 min at 4 °C, followed by centrifuging the supernatant at 10,000*g* for 15 min at 4 °C. The supernatant belonging to the WLC was stored at 4 °C. The supernatant from the sample was loaded onto 400 μl of sucrose solution (60% (w/v), 20 mM Hepes pH 7.5, 1 mM EDTA, 5 mM MgCl_2_, 10 mM KCl, 10% (v/v) glycerol, 100 μg/ml CHX) and centrifuged at 55,000*g* for 2.5 h at 4 °C in an ultracentrifuge (Optima XL Series and Type 70.1 Ti Rotor (Beckman Coulter)). The supernatant was transferred into a fresh tube and the pellet was resuspended in 500 μl of CSB buffer. An equal volume of TRIzol Reagent (Thermo Fisher Scientific 15596026) was mixed with each fraction and the WLC. The RNA was isolated using the TRIzol Reagent in conjunction with the PureLink RNA Mini Kit (Thermo Fisher Scientific 12183018A) as per the manufacturer’s protocol. RNA integrity was checked by resolution on a 1% (w/v) agarose gel and measurement of A260∕A280 nm ratios using a One/One Microvolume UV-Vis Spectrophotometer (Thermo Fisher Scientific). A total of 1 μg of RNA was DNase treated using the Invitrogen TURBO DNase Kit (Thermo Fisher Scientific AM2238). Reverse transcription of RNA was performed using oligo (dT) primers from the Invitrogen SuperScript III First-Strand Synthesis SuperMix (Thermo Fisher Scientific 18080400). No reverse transcriptase controls were also prepared for genomic DNA contamination checks. Two different genomic DNA contamination checks were performed: an intron flanking PCR assay on the newly synthesized complementary deoxyribonucleic acid (cDNA) and the investigative qPCR assays on the no reverse transcriptase controls. Probe-based qPCR assays were designed for GAPDH, NEAT1, DHFR, and DHFR2 using the Universal Probe Library (Roche). Primers and probes are as follows: GAPDH: Forward Primer (FP) 5′CTCTGCTCCTCCTGTTCGAC3′; Reverse Primer (RP) 5′ACGACCAAATCCGTTGACTC3′; Universal Probe (UP) #60. NEAT1: FP 5′AGTGAATGTGCACCCTTGG3′; RP 5′AACAAACCACGGTCCATGA3′; UP #46. DHFR: FP 5′GAACTCGTGACCGCAAGC3’; RP 5′GGCTCAAGCCGGTAATCC3′; UP #75. DHFR2_All: FP 5′AATTTCGCGGCATTCTTG3′; RP 5′GGTTAACACCTCCGAACTTGC3′; UP #72. DHFR_201: FP 5′CGGACCTTAGAAAGTCACACATC3′; RP 5′GCGAAATTCCCTTCTTCAAAT3′; UP #89. DHFR2_202: FP 5′CGTCCAGAAGCGTCTCATTC3′; RP 5′AAGCTCTCAGCGGGACAAT3′; UP #57. All reverse transcription-quantitative polymerase chain reaction (RT-qPCR) reactions were carried out using 1 μl cDNA, the FastStart Essential DNA Probes Master (Roche 06402682001) and the LightCycler 96 system (Roche) as per manufacturers’ protocol. The expression ratios were calculated relative to the target in the WLC sample using the LightCycler 96 qPCR System Instrument Software (Roche; https://sequencing.roche.com/us/en/products/group/lightcycler-96.html).

### Data-dependent Acquisition and Targeted Data Acquisition LC-MS/MS Analysis on a Panel of Human Cell Lines and Tissues

A number of human cell lines and tissues were examined by LC-MS/MS for the presence of the DHFR2 protein. This included the HepG2 cells, IMR32 cells, undifferentiated hiPS cells, NEP cells, hiPS-derived hepatocyte cells, HuH7 cells, placental tissue (Hospital Universitari Sant Joan de Reus––Universitat Rovira i Virgili [HUSJR-URV]), placental membrane tissue (HUSJR-URV), umbilical cord tissue (HUSJR-URV), ovarian tissue (BioIVT 1283091F), and testis tissue (BioIVT 114500A1). The placental and umbilical cord tissues were derived from a control pregnancy from an ongoing study at HUSJR-URV. The ovarian tissue (normal adjacent) was sourced from BIOIVT, Case ID 135341/Specimen ID 128309F, 231 mg; Lot number HMN578647. The human testis tissue (normal adjacent) was also sourced from BIOIVT, Case ID 38773/114500A1/353 mg; Lot number HMN578648. Ethical approval and consent for all human tissues utilized was granted by the Ethics Committee of the Hospital Universitari Sant Joan, Reus and Dublin City University Research Ethics Committee. This work abides by the Declaration of Helsinki principles. The fresh-frozen tissue samples were reduced to a fine powder using a prechilled sterile grinder (Krups F203 Coffee and Spice grinder). Cell lysis (50 μl of powdered tissue or 5 × 10^6^ cells), protein isolation, filter-aided sample preparation (FASP), digestion of protein samples, and purification of enzymatically digested peptides were performed according to (26).

LC-MS/MS analysis was carried out in the Proteomics facility at the National Institute for Cellular Biology, DCU. Data-dependent acquisition (DDA) LC-MS/MS analysis was initially performed on all the digested protein samples. Liquid chromatography (LC) separations were performed using an UltiMate 3000 nanoRSLC system (Thermo Fisher Scientific). One microgram of peptide was loaded onto a trapping column cartridge (PepMap100, C18, 300 μm × 5 mm) (Thermo Fisher Scientific) using 2% (v/v) ACN, 0.1% (v/v) total flow area (TFA) at a flow rate of 25 μl/min for 3 min. The peptides were then resolved on AcclaimPepMap100 75 μm × 50 cm, nanoViper C18, 3 μm, 100 Å (Thermo Fisher Scientific), where a binary gradient was employed; solvent A (2% (v/v) ACN, 0.1% (v/v) formic acid) and 2 to 25% solvent B (80% (v/v) ACN, 0.08% (v/v) formic acid) for 100 min, followed by solvent A and 25 to 50% solvent B for a further 20 min. The column flow rate was 300 nl/min and the column temperature was 35 °C. The peptides were transported into the Orbitrap Fusion Tribrid Mass Spectrometer (Thermo Fisher Scientific), which was set to DDA using a top-speed approach (cycle time of 3 s). A voltage of 2 kV and a capillary temperature of 320 °C was employed for peptide ionisation. Full scans within the 380 to 1500 *m/z* range were carried out in the Orbitrap mass analyser using a resolution of 120,000 (at *m/z* 200), automatic gain control (AGC) target value of 4 × 10^5^, and a maximum ion injection time of 50 ms. The top-speed acquisition algorithm was used to identify the number of selected precursor ions for fragmentation. The Quadrupole selected the precursor ions for fragmentation with an isolation width of 1.6 Da. The peptides with a charge state between 2 + and 6 + were examined and a dynamic exclusion was applied after 60 s. Precursor ions were fragmented using higher energy collision–induced dissociation with a normalised collision energy of 28%. The linear ion trap measured the resulting MS/MS ions under the following scan conditions: AGC target value of 2 × 10^4^ and maximum fill time of 35 ms. Peptides were identified using SEQUEST HT and Percolator through ProteomeDiscover 2.2 (Thermo Fisher Scientific) and either human or mouse Fasta databases from UniprotKB 2021 (mouse contained 36,594 sequences and human contained 20,308 sequences). The following search parameters were used: 20 ppm peptide mass tolerance, 0.6 Da MS/MS mass tolerance, two missed cleavages, fixed modification of cysteine carbamidomethylation and variable modification of methionine oxidation. Only highly confident peptide identifications with a false discovery rate ≤0.01.

A targeted data acquisition (TDA) LC-MS/MS method that targeted a DHFR2-specific peptide (EAMNHLGHLK) was subsequently performed on all the digested protein samples. A synthetic version (AQUA Basic Light from Thermo Fisher Scientific) of the DHFR2 peptide was injected into the LC-MS/MS system and a full MS scan was performed as described in the previous paragraph. The results from this scan were used to generate a TDA LC-MS/MS workflow that would select specific precursor masses within a specific retention time for targeted fragmentation when digested protein samples were separated over a 60 min LC separation using the binary gradient of solvent A and 2 to 25% solvent B for 50 min, and solvent A and 25 to 50% solvent B for a further 10 min. The z = 2 for EAMNHLHGLK peptide had an m/z of 575.297 and a z = 3 of 383.867 m/z had a retention time of 17.4 min. The oxidised form of the peptide (EAM∗HNLHGLK) with z = 2 at 583.293 m/z and its z = 3 of 389.198 m/z had a retention time of 15.2 min. To validate the TDA LC-MS/MS workflow, the DHFR2 synthetic peptide was spiked into 1 μg of enzymatically digested protein sample and loaded onto the UltiMate 3000 RSLCnano System for LC separation. The peptides were separated into the Orbitrap Fusion Tribrid Mass Spectrometer, which was set to TDA using a top-speed approach (cycle time of 3 s). Peptide ionisation and Orbitrap mass analyser initial scan was performed as per previous paragraph, apart from the *m/z* range which was set to 380 to 585. The TDA LC-MS/MS workflow allowed the Quadrupole to select precursor ions for fragmentation with an isolation width of 1.6 Da with an intensity threshold of 5.0E3. A Target Mass selection was used to fragment z = 2575.30 and z = 3383.87 between 16.4 min and 18.4 min and to target z = 2585.3 m/z and z = 3389.2 m/z between 14.7 and 16.2 min. If a precursor ion matched the *m/z*, charge state and retention time window from the target list it would be selected for fragmentation using higher energy collision–induced dissociation with a normalized collision energy of 28%. The Orbitrap mass analyser measured the resulting MS/MS at a resolution of 30,000 (at *m/z* 200) under the following scan conditions: AGC target value of 1 × 10^4^ and maximum fill time of 300 ms. One microgram of the digested protein samples was subsequently analyzed for the endogenous DHFR2 peptide using the optimized TDA LC-MS/MS workflow.

### Proteasome Inhibitor MG132 Treatment on HepG2 Cells for LC-MS/MS Analysis

A T75 flask of HepG2 cells was incubated in DMEM, 10% (v/v) FBS, and 0.005% (v/v) dimethyl sulfoxide (DMSO) (vehicle). A second flask was treated with DMEM, 10% (v/v) FBS, and 10 μM MG132 (Sigma-Aldrich 474790). Both flasks were incubated at 37 °C in a 5% CO2 incubator for 8 h. The cells were harvested, lysed, and protein quantified as per Mammalian Cell Culture and Data-dependent Acquisition and Targeted Data Acquisition LC-MS/MS Analysis on a Panel of Human Cell Lines and Tissues. Western blotting analysis was performed on 75 μg of protein to confirm the build-up of ubiquitinated proteins in the MG132-treated sample compared to the DMSO control. This was performed as per Western Blotting and DHFR Enzyme Activity Assay on HEK293 Mitochondrial Fractions apart from the following steps; the denatured proteins were loaded onto a Novex 4 to 20% Tris-Glycine Mini Gel (Thermo Fisher Scientific XP04202BOX), the membrane was incubated in a primary antibody against Ubiquitin (Thermo Fisher Scientific 14607882) at a concentration of 2 μg/ml, followed by an anti-mouse HRP-conjugated secondary antibody, and the SuperSignal West Pico PLUS Chemiluminescent Substrate (Thermo Fisher Scientific 34580) was used to visualize the target proteins. The remaining protein samples were prepared for LC-MS/MS analysis according to Data-dependent Acquisition and Targeted Data Acquisition LC-MS/MS Analysis on a Panel of Human Cell Lines and Tissues and analyzed using the LC-MS/MS methods described in Data-dependent Acquisition and Targeted Data Acquisition LC-MS/MS Analysis on a Panel of Human Cell Lines and Tissues.

### Multiple Reaction Monitoring LC-MS/MS Analysis on Human Embryonic and Foetal Tissue

This experiment was carried out in the nervous system development and translational mass spectrometry research laboratories at Great Ormond Street Institute of Child Health, University College London. Human embryonic and fetal material was provided by the Joint MRC/Wellcome Trust (Grant # MR/006237/1) Human Developmental Biology Resource (http://www.hdbr.org). The HDBR (Human Developmental Biology Resource) sample collection is largely from within the legal termination period in the UK. Three developmental stages (early, mid, and late sample collection time points) were selected to represent a range of stages that was available for this study as it is not known when/where DHFR2 is expressed. The selection was also based on the range of different tissue types available for each embryo to allow comparisons between the stages. Ethical approval was provided by the Fulham Research Ethics Committee (18/LO/022; HDBR UCL) and the North East-Newcastle & North Tyneside 1 Research Ethics committee (18/NE/0290; Newcastle University). This work abides by the Declaration of Helsinki principles.

Human embryonic and fetal tissue was examined for the presence of the DHFR2 protein by LC-MS/MS. This included the kidney, heart, lung, liver, and brain tissue from the Carnegie stage 21 to 22, postconceptual week 9 to 10 and postconceptual week 15 to 17 (Human developmental Biology Resource, supplemental Table S1). Cell lysate was isolated from approximately 100 μg of tissue using 200 μl of lysis buffer (100 mM ammonium bicarbonate, 100 mM DTT, 1% (w/v) sodium deoxycholate, pH 8), the tissue homogenizing CKMix (Precellys P000918-LYSK0-A), and the Minilys personal homogenizer (Bertin Technologies) under the highest speed for 10 s for three runs. Cellular debris was removed by centrifuging the lysate at 16,000*g* at room temperature for 15 min. The proteins were denatured by incubating the supernatant at 85 °C for 20 min. The proteins were alkylated by adding 50 μl of 600 nM iodoacetamide and incubated in a dark place for 20 min. The proteins were enzymatically digested by adding 2 μg of Trypsin Gold (Promega V5280) and incubating at 37 °C overnight. The samples were acidified by adding 5 μl of 6% (v/v) of TFA and rotated for 10 min. The peptides were centrifuged at 16,000*g* at room temperature for 10 min. A solid-phase extraction cleanup was performed on the supernatant using the Isolute C18 cartridges (Biotage 220-0010-A). The cartridges were solvated by adding 1 ml of 60% (v/v) ACN, 0.1% (v/v) TFA. The solution was left to fully pass through the column; this was performed for subsequent steps. The column was equilibrated by adding 1 ml of 0.1% (v/v) TFA and this step was repeated. The trypsinized peptide samples were loaded onto the column. The bound peptides were washed by adding 1 ml of 0.1% (v/v) TFA to the column. The peptides were eluted by adding 500 μl of 60% (v/v) ACN, 0.1% (v/v) TFA to the column. A concentrator plus (Eppendorf) was used to dry down the samples.

A multiple reaction monitoring (MRM) LC-MS/MS workflow was created on SkyLine to target tryptic peptides that were unique to the DHFR2 protein. The workflow was validated by running a trypsinized whole protein lysate from HepG2 cells containing the recombinant DHFR2 protein (generated from the pCMV6-AC-DHFR2-tGFP expression vector [OriGene RG232027]). This sample was prepared according to Data-dependent Acquisition and Targeted Data Acquisition LC-MS/MS Analysis on a Panel of Human Cell Lines and Tissues. The lyophilized samples were resuspended in 100 μl of 5% (v/v) ACN, 0.1% (v/v) TFA, and rotated at room temperature for 10 min at 1000 rpm to ensure the trypsinized peptides were fully resuspended. They were then centrifuged at 16,000*g* at room temperature for 10 min, and the supernatant containing the peptides were separated on ACQUITY ultra performance liquid chromatography peptide CSH C18 Column (Waters). The dimension of the column (Cat. no. 186006938) was 130 Å (pore size), 1.7 μm (particle size), 2.1 mm (inner diameter) X 150 mm (length), 1K to 15K (molecular weight range). Solvent A was 0.1% (v/v) formic acid and solvent B was ACN, 0.1% (v/v) formic acid. The ultra performance liquid chromatography gradient starting settings was 95% solvent A, 5% solvent B, followed by 95% solvent A, 5% solvent B for 1 min; 80% solvent A, 20% solvent B for 4.5 min; 70% solvent A, 30% solvent B for 9.8 min; 100% solvent B for 10.5 min; 100% solvent B for 11.99 min; 95% solvent A, 5% solvent B for 12 min; 95% solvent A, 5% solvent B for 15 min. The separation used a flow rate of 0.15 ml/min and a column temperature of 50 °C. The peptides were eluted into the Xevo TQ-S Tandem Quadrupole Mass Spectrometer (Waters) for MRM detection in positive-ion mode. The spray voltage was set to 2.8 kV, the desolvation temperature 400 °C, the cone gas flow rate 150 L/h and desolvation gas flow rate at 600 L/h. The collision gas consisted of nitrogen and was set at 0.25 ml/min. The nebulizer operated at 7 bar. The cone energy was set to 25 V and the collision energies varied depending on the optimal settings for each peptide. Chromatograms were analyzed using the SkyLine software (https://sciex.com/products/software/skyline-software). The MRM LC-MS/MS workflow was adapted to only include the DHFR2-specific peptides identified in the validation step and internal control peptides. The human embryonic and fetal peptide mixtures were assessed using the adapted MRM LC-MS/MS method using the settings described above (resuspended in 50 μl of 5% (v/v) ACN, 0.1% (v/v) TFA).

### Experimental Design and Statistical Rational

The DDA and TDA analyses described in Data-dependent Acquisition and Targeted Data Acquisition LC-MS/MS Analysis on a Panel of Human Cell Lines and Tissues included tissue samples from one individual or biopsy and the cell line samples were from one flask. Duplicate samples were prepared for each tissue or cell line and analyzed in the same run. Each sample was run once through the instrument. The control for the Proteasome inhibitor MG132 treatment on HepG2 cells for LC-MS/MS analysis was the HepG2 cells plus DMSO (vehicle). A synthetic peptide or recombinant DHFR2 protein was used during method development and quality control checks for the TDA analysis. As this was a qualitative analysis, no statistical analysis was performed.

For the analyses described in Multiple Reaction Monitoring LC-MS/MS Analysis on Human Embryonic and Foetal Tissue, individual tissue types for each stage were from different embryos (supplemental Table S1). A single sample was prepared and run for each tissue type and stage. Each sample was run once through the instrument. A recombinant DHFR2 protein was used during method development and quality control checks for the MRM analysis. As this was a qualitative analysis, no statistical analysis was performed.

## Results

### Purified Mitochondria from Human Cell Lines Demonstrate Mitochondrial DHFR Activity That is Derived From DHFR and Not DHFR2

**Fig. 1:**
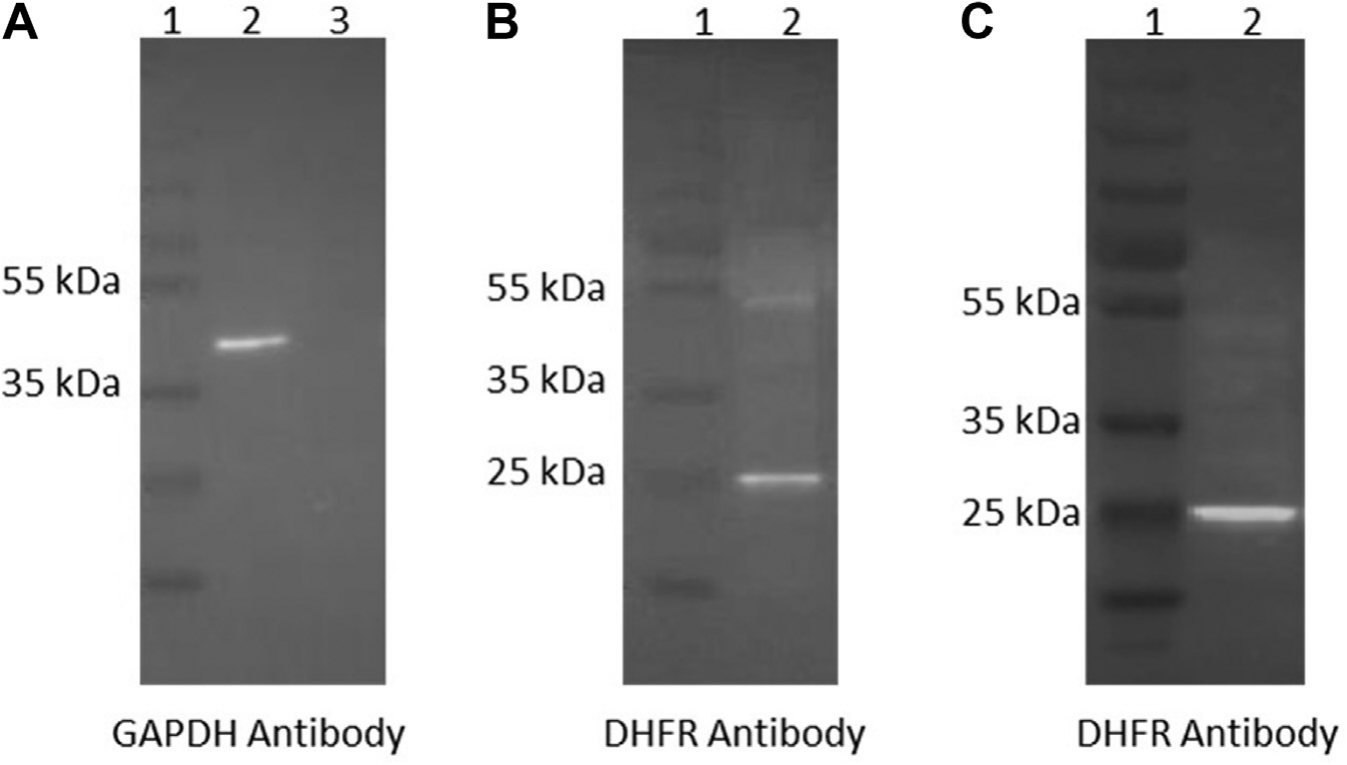
**Western blot analysis confirms the dihydrofolate reductase protein in the mitochondria of HEK293 cells.***A*, purity of the cytoplasmic and mitochondrial fractions confirmed by probing blot with a GAPDH antibody. The cytoplasmic control GAPDH was only identified in the cytoplasmic protein fraction at 36 kDa. *Lane 1*, 180 kDa protein ladder; *Lane 2*, HEK293 cytoplasmic protein fraction; and *Lane 3*, HEK293 mitochondrial protein fraction. *B*, DHFR protein identified at 21 kDa in cytoplasmic fraction probed with DHFR antibody. A higher molecular weight band was also detected at approximately 55 kDa. *Lane 1*, 180 kDa protein ladder and *Lane 2*, HEK293 cytoplasmic protein fraction. *C*, DHFR protein identified in mitochondrial fraction probed with DHFR antibody. *Lane 1*, 180 kDa protein ladder and *Lane 2*, HEK293 mitochondrial protein fraction.

We purified mitochondrial protein fractions from HEK293 cells and assessed endogenous DHFR activity using a colorimetric enzyme activity assay. A high level of reductase activity was detected in the HEK293 mitochondrial fraction. A true ΔOD/min of 0.009 and a specific activity of 14.88 nmol/min/mg were calculated for this fraction (supplemental Fig. S1). Furthermore, Western blot analysis probed with a DHFR antibody confirmed the presence of the protein in the cytoplasmic and mitochondrial fractions (Fig. 1). It was vital that the purity of the mitochondrial fraction was examined as other cellular compartments contain the DHFR enzyme. Therefore, cytoplasmic fractions were isolated as a control for immunoblotting. Western blot analysis detected GAPDH protein (cytoplasmic positive control) only in the cytoplasmic fractions, confirming no cross compartment contamination in the mitochondrial purification protocol (Fig. 1). These results confirm that there is endogenous DHFR activity present in the mitochondria, which correlates with previous analyses (4, 27). We also note a higher molecular weight band at around the 55 kDa mark in the cytoplasmic fraction (Fig. 1*A*) of our Western blot when probed with an anti-DHFR antibody. We observed this consistently in other Western blot experiments (data not shown). The known localization of DHFR in the cytoplasm and nucleus of cells (5, 28) and the likely crossreactivity of commercial antibodies to DHFR and DHFR2 made it necessary to purify mitochondrial fractions as a means to detect natural endogenous DHFR2. Mitochondrial protein fractions were extracted from HepG2 cells and proteins between 18 to 24 kDA were isolated using an SDS-PAGE gel extraction technique (supplemental Fig. S2). DHFR and DHFR2 are predicted to be of similar size, that is, 21 kDa. Nano LC-MS/MS analysis was used to identify peptides within these fractions. Strict settings were selected to analyze the raw data, including detecting peptides that may have been subject to any modifications, including oxidation on methionine or carbamidomethylation on cysteine. This analysis on the isolated samples detected a peptide that was common to both the DHFR and DHFR2 protein meaning we could not eliminate the presence of either protein from this peptide identification. The instrument did not recognize any peptide that was exclusive to the DHFR2 protein; however, it did detect five tryptic peptides that were unique to DHFR protein resulting in a sequence coverage of approximately 30% (supplemental Table S2, supplemental Figs. S3, and S4; SI DDA peptide identification mitochondria 21 kDa gel extraction analysis). This data provides compelling evidence that DHFR (and not DHFR2) is the reductase activity in human mitochondria.

### DHFR2 RNA Isoforms Show Differential Association With the Ribosome

Published Ribo-seq data from the RPFdb v2.0 beta platform was utilized to assess the relationship between DHFR2 RNAs and the ribosome. Prior to examining the DHFR2 RNA, quality control checks were performed by examining the RPKM values for the *GAPDH, POLG*, *MTHFD1*, *TYMS*, and *DHFR* genes. Outliers within the ubiquitously expressed controls (*GAPDH* and *POLG*) were excluded from subsequent analyses. The *MTHFD1, TYMS* and *DHFR* protein-coding genes were used as ribosome-bound OCM controls. As expected, the Ribo-seq data revealed that the RNA belonging to these genes bind to the ribosome. And finally, the RPKM values for the *DHFR2* gene revealed that the DHFR2 RNA binds to the ribosome in all samples (supplemental Fig. S5).

**Fig. 2:**
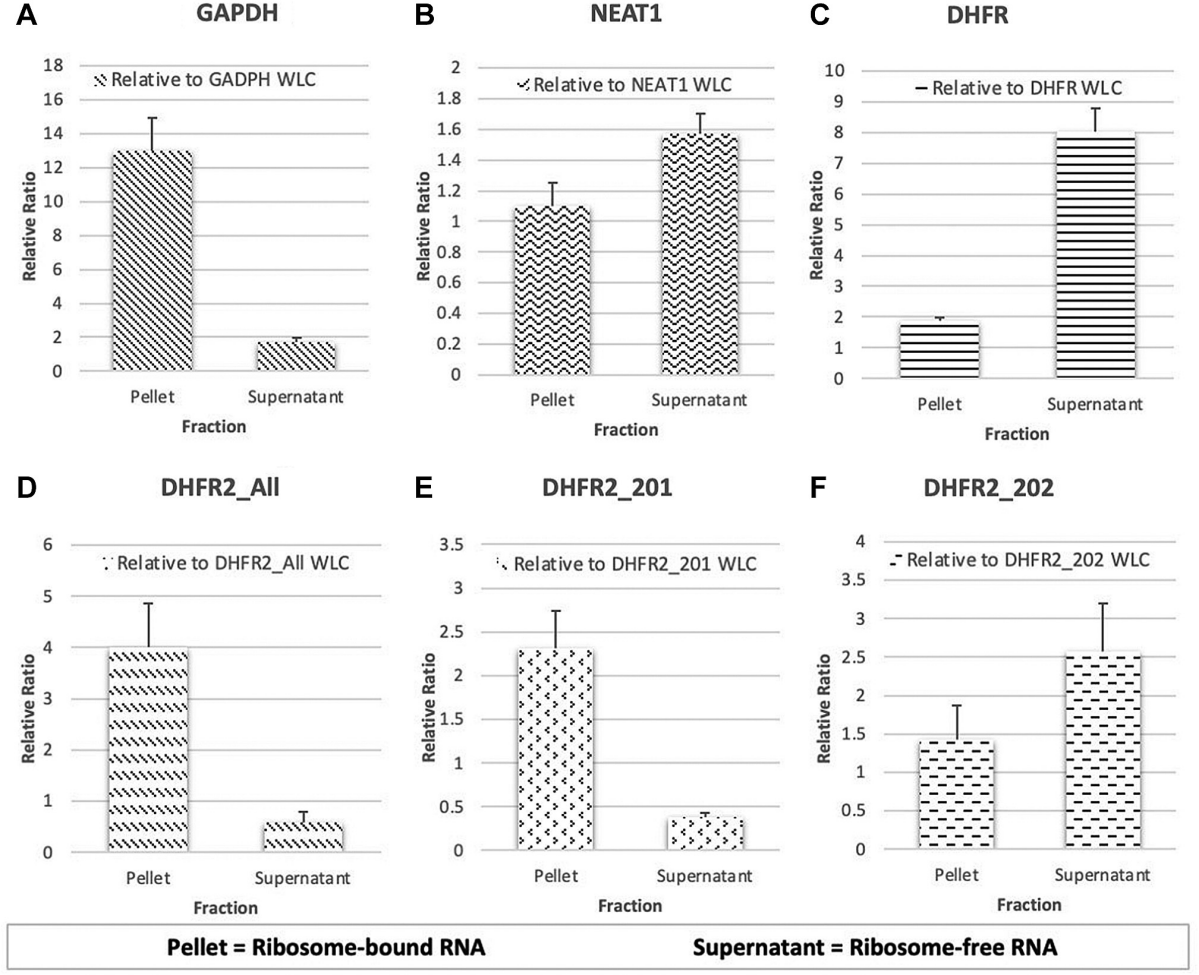
**Sucrosecushion ultracentrifugation RT-qPCR reveals the main DHFR2 transcripts show differential association with the ribosome.** Six RT-qPCR assays were performed on the RNA fractions from the sucrose cushion ultracentrifugation and the RNA WLC. The expression ratios of the target RNA in the pellet and supernatant fractions were calculated relative to the target in the WLC. *A*, GAPDH (ribosome-bound control) was more abundant in the ribosome-bound RNA fraction. *B*, NEAT1 (ribosome-free control) was present at a higher concentration in the ribosome-free RNA fraction. *C*, Dihydrofolate reductase (DHFR) gene family control was present at a higher concentration in the ribosome-free RNA fraction. *D*, the DHFR2_All assay showed that the DHFR2 RNA was more abundant in the ribosome-associated RNA fraction. *E*, DHFR2_201 present at a higher concentration in the ribosome-bound RNA fraction. *F*, DHFR2_202 slightly more abundant in the ribosome-free RNA fraction. RT-qPCR, reverse transcription-quantitative polymerase chain reaction; WLC, whole-lysate control.

A sucrose cushion ultracentrifugation RT-qPCR technique was employed to examine the relationship between the ribosome and individual DHFR2 transcripts (DHFR2_201 and DHFR2_202). This enrichment technique generated three RNA samples: a pellet fraction (ribosome-bound RNA), a supernatant (ribosome-free mRNA) fraction and a WLC. Steps were taken to confirm the integrity of the RNA and that the cDNA was free from genomic deoxyribonucleic acid. Six qPCR assays (GAPDH, NEAT1, DHFR, DHFR2_All, DHFR2_201, and DHFR2_202) were performed on the quality-controlled cDNA. The GAPDH, NEAT1, and DHFR assays were experimental controls. *GAPDH* is a protein-coding gene and therefore was employed as the ribosome-bound control; as expected, its RNA was more abundant in the ribosome-bound RNA fraction (Fig. 2*A*). NEAT1 is a long noncoding RNA and was the ribosome-free control; as predicted, its RNA was present at a higher concentration in the ribosome-free RNA fraction (Fig. 2*B*). *DHFR* is the main protein-coding gene in the DHFR gene family and therefore was the reductase gene family control; unexpectedly, its RNA was more abundant in the ribosome-free RNA fraction (Fig. 2*C*). An assay that targets all the DHFR2 transcripts was performed to confirm the results obtained from the Ribo-seq data; the DHFR2_All RNA was more abundant in the ribosome-associated RNA fraction (Fig. 2*D*). Finally, two assays were designed to target the two main DHFR2 RNA isoforms, DHFR2_201, and DHFR2_202. These qPCR assays revealed the DHFR2_201 transcript was significantly more abundant in the ribosome-bound RNA (Fig. 2*E*), whereas DHFR2_202 was slightly more abundant in the ribosome-free RNA fraction (Fig. 2*F*). This result demonstrates that the main DHFR2 RNA isoforms show differential association with the ribosome.

### The DHFR2 Protein was Not Detected in a Panel of Human Cell Lines and Tissues

**Fig. 3:**
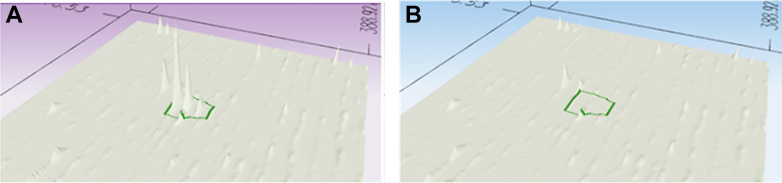
**Three-dimensional distribution plots generated from the TDA LC-MS/MS analysis on the undifferentiated hiPS sample.***A*, the isotopic pattern shown for the cell lysate spiked with the synthetic version of the DHFR2-specific peptide. An MS/MS response occurred, identifying a normal and posttranslationally modified version of the synthetic peptide. *B*, the isotopic pattern for the cell lysate. No MS/MS response occurred, meaning no version of the endogenous DHFR2 peptide was detected in this sample. DHFR, dihydrofolate reductase; hiPS, human-induced pluripotent stem; MS/MS, tandem mass spectrometry; TDA, targeted data acquisition.

The proteome of multiple cell and tissue types was examined for peptides unique to the DHFR2 protein. Total protein was isolated from HepG2 cells, IMR32 cells, undifferentiated hiPS cells, NEP cells, hiPS-derived hepatocytes, HuH7 cells, placental tissue, placental membrane tissue, umbilical cord tissue, ovaries tissue, and testis tissue. DDA LC-MS/MS analysis was carried out on the enzymatically digested protein lysate from these samples. Peptides common to the DHFR and DHFR2 proteins were identified in the HepG2, IMR32, undifferentiated hiPS, NEP, and HuH7 samples. It was not possible to detect a peptide with a sequence exclusive to the DHFR2 protein in any of the samples but DHFR-specific peptides were detected (supplemental Table S3). This data suggests that the peptides identified in these samples that were shared by both proteins belonged to the DHFR protein. Therefore, the DDA LC-MS/MS analysis (SI DDA Results Tables for the panel of human cell lines and tissues) found no evidence of the DHFR2 protein in any of these cell lines and tissues. It was not possible to detect by LC-MS/MS any DHFR peptides in the hiPS hepatocyte cells, placental tissue, placental membrane tissue, umbilical cord tissue, and ovaries tissue; even though other OCM proteins were identified, including MTHFD1, MTHFD1L, MTHFD2, SHMT1, and SHMT2. The lack of detection of DHFR in these samples could imply that the DDA LC-MS/MS analysis was not sufficiently sensitive for this type of analysis. Thus, a more sensitive TDA LC-MS/MS method was employed to interrogate these samples further for a preselected DHFR2-specific peptide (EAMNHLGHLK). A synthetic version of the DHFR2 peptide was run by LC-MS/MS to determine the *m/z*, charge state, and retention time of the nonmodified and any posttranslationally modified versions of the peptide. The data obtained was used to create a workflow on the instrument to only identify peptides with these specific characteristics. The workflow was validated by running an enzymatically digested whole-protein lysate spiked with the synthetic peptide (Fig. 3*A*). The enzymatically digested protein lysate from the cell lines and tissues was assessed using the TDA LC-MS/MS workflow. There was no MS/MS response triggered for the samples (Fig. 3*B*), indicating that the endogenous version of the DHFR2 peptide was not detected in any of the cell lines and tissues tested. Therefore, this analysis found no evidence that the *DHFR2* gene encodes a functional protein in the adult cell lines and tissues examined.

### Ubiquitination is Not Driving DHFR2 Protein Degradation Leading to Nondetection

HepG2 cells treated with the MG132 proteasome inhibitor were examined for DHFR2-specific peptides to determine if the DHFR2 protein is being degraded *via* the ubiquitin-proteasome pathway. Western blotting against ubiquitin confirmed a build-up of ubiquitinated proteins in the MG132-treated cells compared to the DMSO control (supplemental Fig. S6). DDA LC-MS/MS analysis on the MG132-treated cells detected a peptide that was common to both the DHFR and DHFR2 protein (supplemental Table S4). The instrument did not recognize any peptide that was unique to the DHFR2 protein but did detect five tryptic DHFR-specific peptides. This data suggests that the peptide identified in this sample that was shared by both proteins belonged to the DHFR protein. This is consistent with the nondetection of the endogenous DHFR2 protein in the MG132-treated sample. Similar results were observed when the TDA LC-MS/MS strategy was performed on the cells treated with MG132. Therefore, this analysis found no evidence that the DHFR2 protein is prone to degradation *via* the ubiquitin–proteasome pathway.

### A DHFR2-Specific Peptide Detected Only in Human Embryo and Fetal Heart Tissue

**Fig. 4:**
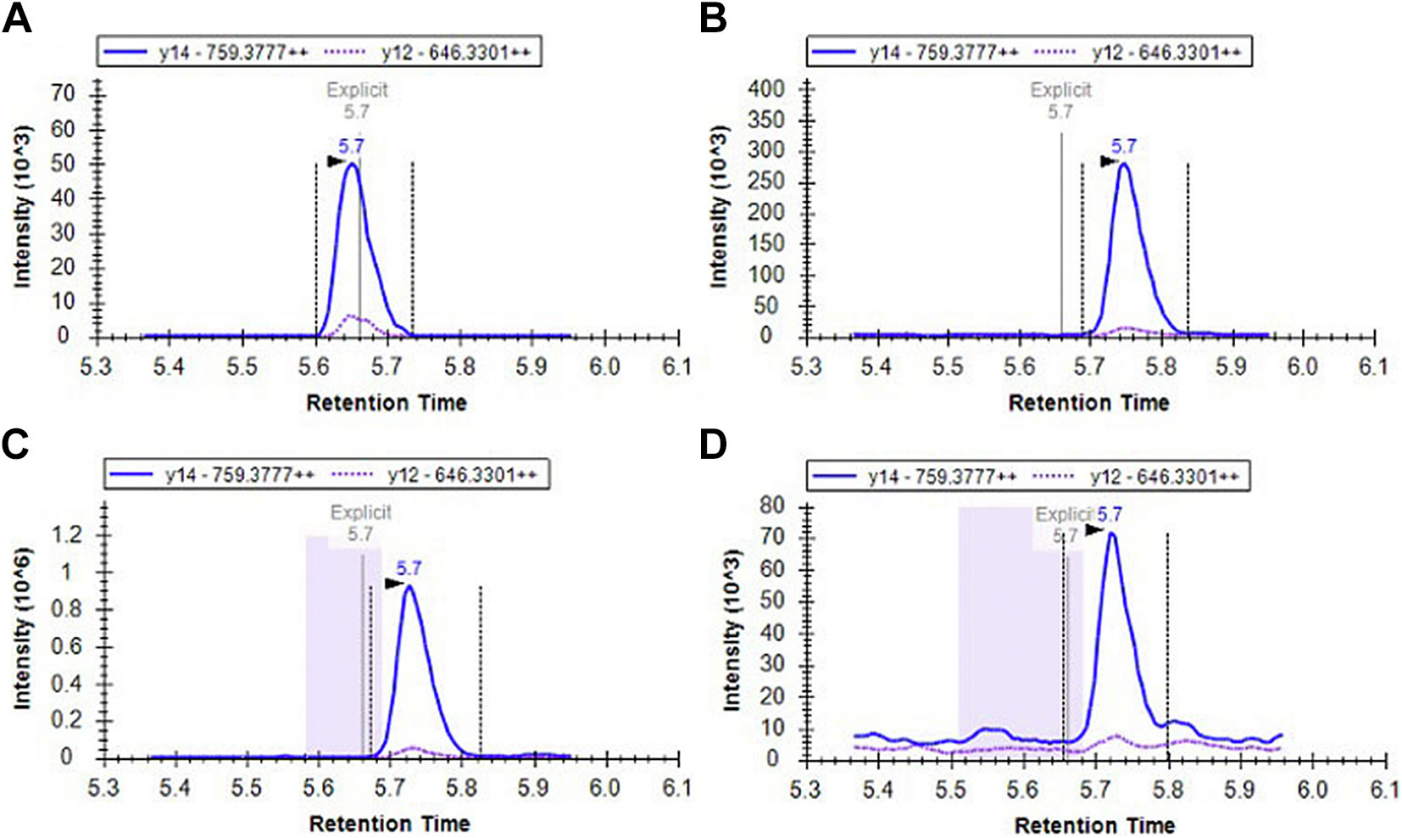
**The DHFR2-specific peptide LLPEYPGVLSDVQEGK detected in human embryo and fetal heart tissue.** Human embryonic and fetal tissue was examined for the presence of the DHFR2 protein by LC-MS/MS. This figure shows the chromatogram results of the DHFR2 peptide LLPEYPGVLSDVQEGK in (*A*) whole-protein lysate containing the recombinant DHFR2 protein (control), (*B*) Carnegie stage 21 to 22 heart tissue, (*C*) postconceptual week 9 to 10 heart tissue, and (*D*) postconceptual week 15 to 17 heart tissue. The peak shown shows the final two optimal transitions that are used as quantifier and qualifier ions. Each peptide in the assay was run with two transitions, one quantifier (*solid line*) and qualifier (*dotted line*). Correct identification was obtained using a digest of recombinant proteins to determine peptide fragmentation profile and establish retention times for each of the targets (supplemental Fig. S7). The peptide peak also elutes at the expected retention time of 5.7 min according to hydrophobicity. This result suggests that the LLPEYPGVLSDVQEGK peptide was detected in the heart tissue lysate from Carnegie stage 21 to 22, postconceptual week 9 to 10, and postconceptual week 15 to 17. DHFR, dihydrofolate reductase.

The proteome of human embryonic and foetal tissue was examined for peptides exclusive to the DHFR2 protein. Prior to examining the human embryonic/fetal tissue, the MRM LC-MS/MS workflow that targeted the eight DHFR2-specific tryptic peptides had to be validated. The transition data is available as supplementary file “SI Transitions Data Table.” Four of the DHFR2 peptides were identified when analyzing a trypsinized whole-protein lysate containing the recombinant DHFR2 protein using the workflow; NGDLPRPPLR, QNLVIMGR, VDMIWIVGGSSVYK, and LLPEYPGVLSDVQEGK. The successful detection of the recombinant DHFR2 protein deemed the MRM LC-MS/MS workflow capable of identifying the endogenous DHFR2 protein in protein lysate sample from human embryonic tissue. The MRM LC-MS/MS workflow was adapted to only include these four peptides and internal control peptides (cytoplasmic (ACTB and TUBA1A), nucleic (LMNB1), and mitochondrial (HSPD1, COX4I1, and TOM20)). The kidney, heart, lung, liver, and brain tissue from the three different stages of human development were assessed using the adapted MRM LC-MS/MS workflow. All the tissues had at least one of the cytoplasmic, nucleic, and mitochondrial internal control peptides present (supplemental Table S5); meaning the sample preparation was successful. The NGDLPRPPLR, QNLVIMGR, and VDMIWIVGGSSVYK DHFR2 peptides were not identified in any of the human embryonic and fetal tissues (supplemental Table S5; SI MRM analysis for human embryonic and fetal tissue). The LLPEYPGVLSDVQEGK peptide was also not detected in the kidney, lung, liver, and brain tissue from the three developmental stages (supplemental Table S5). There was, however, evidence that the LLPEYPGVLSDVQEGK DHFR2-specific peptide was present in the heart tissue from the Carnegie stage 21 to 22, postconceptual week 9 to 10 and postconceptual week 15 to 17 samples (Fig. 4 and supplemental Table S5; fragmentation mass spectrum supplemental Fig. S7). The detection of this peptide in this tissue suggests that the endogenous DHFR2 protein is expressed in heart tissue during embryo and fetal development.

## Discussion

We demonstrate that while purified mitochondria derived from a human HEK293 cell line contains DHFR enzyme activity, this activity arises from the DHFR and not the DHFR2 enzyme (Fig. 1, supplemental Figs. S1, S2, and supplemental Table S2). This mitochondrial reductase activity is likely to support *de novo* thymidylate synthesis in mitochondria, as reported originally (4). While this evidence rules out DHFR2 as being the mitochondrial reductase enzyme, it does not preclude the potential protein from having a functional role in other aspects of the cell. We then sought experimental evidence that the DHFR2 ORF was in fact translated.

We assessed the translational capabilities of endogenous *DHFR2* mRNA isoforms using TDA LC-MS/MS analyses across human-derived cell lines and tissue types, in combination with an assessment of their association with the ribosome. TDA LC-MS/MS was performed on available laboratory cell lines and cell lines/tissues that have relevance during development. The latter focus was driven by the possible association of *DHFR2* with risk of neural tube defects in an Irish cohort (29). The cell lines included HEK293, HepG2, undifferentiated hiPS, HuH7, hiPS-derived hepatocytes, IMR32, and NEP. The human tissues examined included placenta, placental membrane, umbilical cord, ovaries, testis, and embryo of various stages. Our detailed proteomic analysis has confirmed that despite a relative abundance of mRNA isoforms arising from the *DHFR2* gene (4, 22); https://www.proteinatlas.org/ENSG00000178700-DHFR2/summary/rna), they do not appear to be translated into the most obvious ORF, that is, a DHFR enzyme in all but one of the tissue types that we examined. We considered, but did not detect, several posttranslational modifications (oxidation on methionine or carbamidomethylation on cysteine) of DHFR2 to account for nondetection of DHFR2-specific peptides. We also demonstrate that endogenous DHFR2 is unlikely to be subjected to ubiquitination and rapidly degraded by the ubiquitin–proteasome pathway (supplemental Table S4). Our ribosome association analyses using an experimental (Fig. 2) and bioinformatics approach (supplemental Fig. S5) indicate that two of the DHFR2 mRNA isoforms, that is, 201 and 202 (as annotated by Ensembl) show differential association with the ribosome. It is possible that at least one RNA isoform is retained in the nucleus supported by our direct evidence of RNA editing by Sanger sequencing (data not shown) and the presence of the required consensus sequence elements required for nuclear retention as reported by Chen *et al.* (30).

Our combined data implies that although at least one *DHFR2* mRNA isoform preferentially associates with the ribosome and is an indicator of translational capability, however, our in-depth proteomics analysis suggests that translation does not occur at a level that is sufficient to allow detection in most cells and tissues. In addition, the identification of DHFR as the mitochondrial reductase enzyme that is likely to support *de novo* thymidylate synthesis in that compartment, suggests that the *DHFR2* gene may have functional significance beyond that of a simple reductase enzyme. The nondetection of DHFR2-specific peptides was also true of several human embryonic tissues except heart. One out of a total of eight unique DHFR2 peptides was detected in all three developmental stages of the heart tissue (Fig. 4). The detection of only one peptide from a protein is common when the protein of interest is present at an extremely low abundance (31, 32). Further investigation will be required to confirm this finding and to elucidate the functional significance of this, but it does indicate that the translation of DHFR2 mRNA only occurs during embryogenesis and may have a specific functional role in the developing heart. To speculate as to what that specific role might be, it may relate to tetrahydrobiopterin (BH4) recycling, where dihydrobiopterin is reduced to BH4 in endothelial cells. BH4 is required for nitric oxide synthesis and an adequate supply may prevent the generation of superoxide release *via* uncoupling of endothelial nitric oxide synthase (33). BH4 recycling has been previously assigned as a secondary activity of DHFR in adult endothelial cells (34); but the affinity of human DHFR for this substrate was subsequently reported as being quite low (35). The embryonic-specific DHFR2 protein may be a more optimal enzyme for this function during a critical window of embryogenesis to support vascular normalization. However, several future lines of investigation will be required to assess this.

In conclusion, our data shows that the main function of the human *DHFR2* gene is not to produce a translated enzyme product in adult cells and that the DHFR activity of the mitochondria can be definitely assigned to DHFR. The main functionality of *DHFR2* is likely to arise from its many RNA isoforms.

## Data availability

All data files in relation to this manuscript are available as supplementary files and at:

MassIVE database: ftp://MSV000092045@massive.ucsd.edu, https://doi.org/10.25345/C5222RG7B

Panorama database: https://panoramaweb.org/dhfr2.url

## Supplemental data

This article contains supplemental data.

## Conflict of interest

The authors declare no competing interests.

## Supplementary Data

SI DDA Results panel of human cell lines and tissues

SI DDA peptide identification Mitochondria 21kDa Gel

SI DHFR2 Translation Paper 2024 REVISED FINAL

SI MRM Analysis for human embryonic and foetal tissue

SI Transitions Data Table
