# Supplementary material for: The Differential Translation Capabilities of the Human DHFR2 Gene Indicates a Developmental and Tissue-Specific Endogenous Protein of Low Abundance
Source: Mol Cell Proteomics. 2024 Jan 14;23(3):100718. doi: 10.1016/j.mcpro.2024.100718 (PMC10884974; doi:10.1016/j.mcpro.2024.100718)
Supplement: SI DHFR2 Translation Paper 2024 REVISED FINAL [file mmc3.docx]

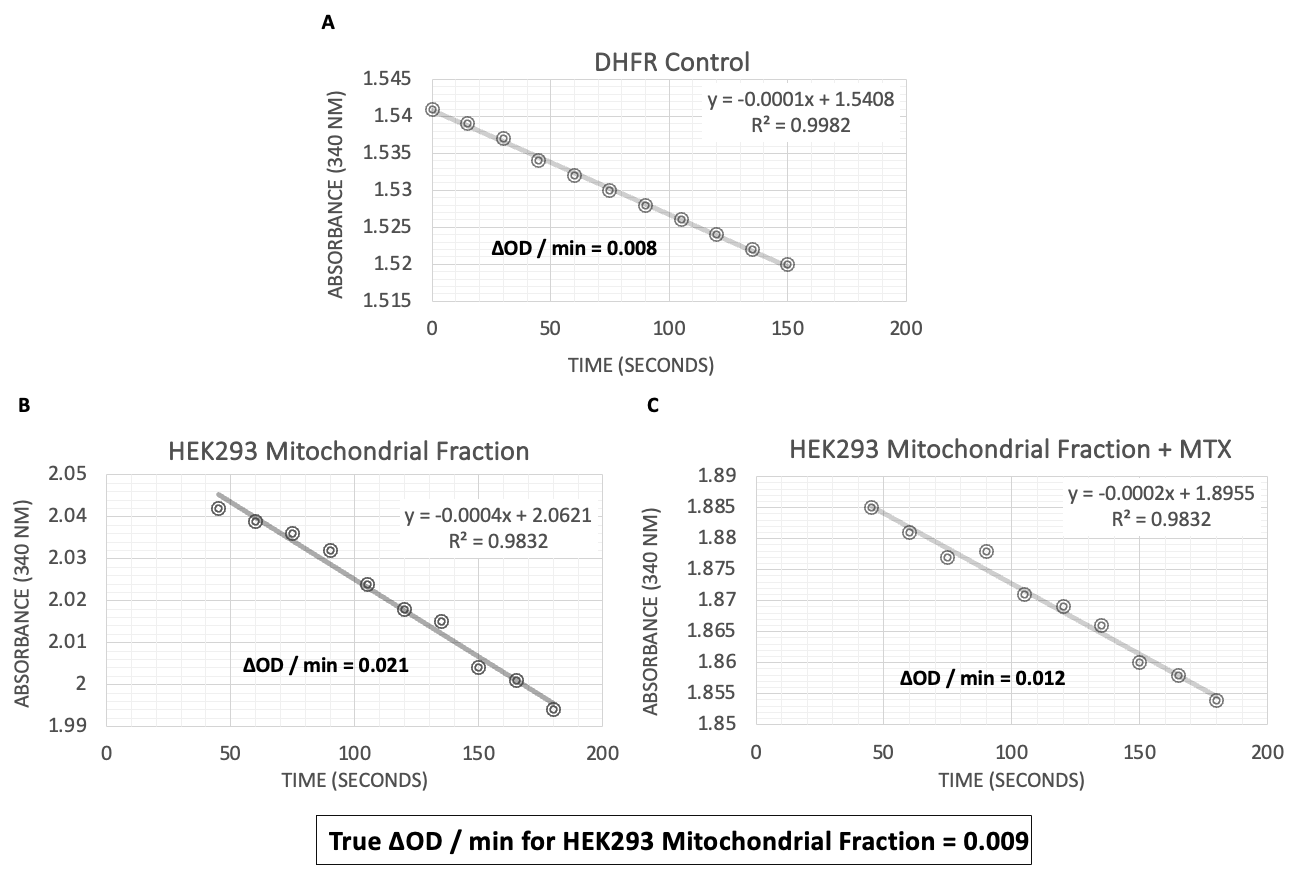


**Figure S1. Dihydrofolate reductase activity detected in the purified mitochondrial fractions.** Mitochondria were purified from HEK293 cells and assessed for dihydrofolate reductase activity using a colorimetric assay that measures loss of absorbance of the cofactor NADPH. (A) Human recombinant DHFR protein was assessed as a positive control for the assay. Enzymatic activity was detected as shown by the decrease in absorbance (ΔOD / min = 0.008). (B) Enzymatic activity detected in the HEK293 mitochondrial fraction, indicated by the decrease in absorbance over time (ΔOD / min = 0.021). (C) A decrease was also shown in the HEK293 mitochondrial fraction plus methotrexate, ΔOD / min of 0.012. Methotrexate inhibits DHFR activity therefore this value is considered the background activity present in the mitochondrial samples. Once the background noise was subtracted from the HEK293 mitochondrial fraction it gave true ΔOD / min of the HEK293 mitochondrial fraction. The true ΔOD / min of 0.009 indicated that the HEK293 mitochondrial fraction had reductase activity.


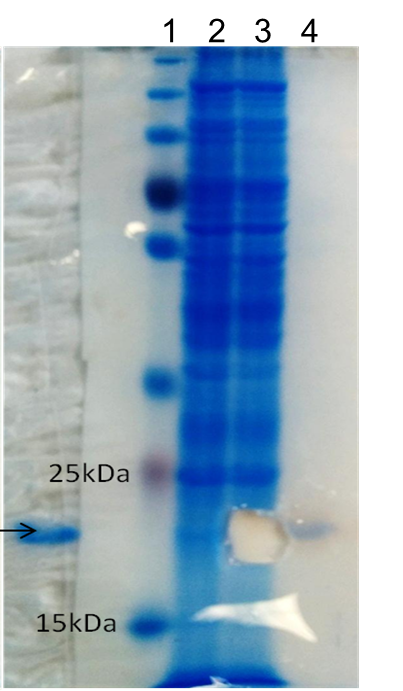


**Figure S2.** **Gel extraction and purification of proteins between 18 - 24 kDa from HepG2 mitochondrial fractions for LC-MS/MS analysis.** The entire isolated mitochondrial sample (2, 3) were resolved on a 12% SDS-PAGE gel overnight at 4 °C and subsequently stained using Coomassie Brilliant Blue G250. The 15 to 180 kDa Protein Ladder (1) was used as a reference to cut out one large band around 21 kDa for LC-MS/MS analysis (3). This band was divided into three pieces (Band 1-3) and sent to Alphalyse for Q-ToF Nano LC-MS/MS analysis.

Table S1. Breakdown of each of the human embryonic/foetal tissues

| **Stage** | **Tissue** | **Human embryo number** | **Aliquot number** |
| --- | --- | --- | --- |
| Carnegie stage 21-22 | Kidney | 12613 | 5 |
|  | Heart | 14755 | 5 |
|  | Lung | 11364 | 10 |
|  | Liver | 12092 | 15 |
|  | Brain | 12210 | 3 |
| Post-conceptual week 9-10 | Kidney | 10951 | 5 |
|  | Heart | 14326 | 9 |
|  | Lung | 12412 | 10 |
|  | Liver | 12542 | 8 |
|  | Brain | 12543 | 23 |
| Post-conceptual week 15 -17 | Kidney | 14104 | 8 |
|  | Heart | 14559 | 9 |
|  | Lung | 13209 | 57 |
|  | Liver | 13454 | 22 |
|  | Brain | 13008 | 12 |

**Table S2. Dihydrofolate reductase peptides identified from the Nano LC-MS/MS analysis on the HepG2 mitochondrial protein fraction**

| **Peptide Sequence** | **Modifications** | **Protein Name** |
| --- | --- | --- |
| INLVLSR | - | DHFR / DHFR2 |
| ELKEPPQGAHFLSR | - | DHFR |
| ImQDFESDTFFPEIDLEK | Oxidation (M) | DHFR |
| ImQDFESDTFFPEIDLEKYK | Oxidation (M) | DHFR |
| LLPEYPGVLSDVQEEK | - | DHFR |
| LLPEYPGVLSDVQEEKGIK | - | DHFR |


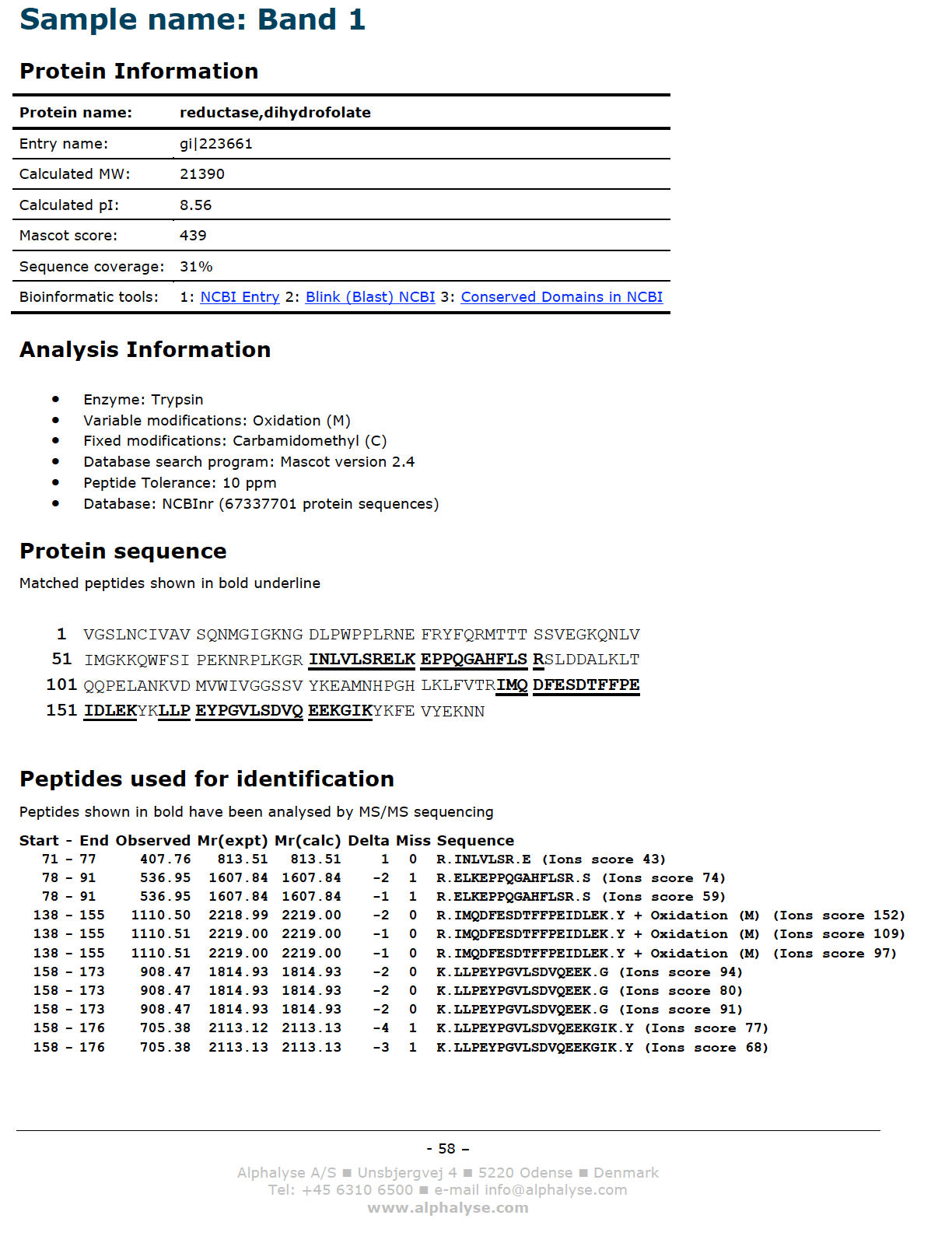


**Band 1**

Peptide 71-77

DHFR2         IPEKNRPLKDRINLVLSRELKEPPQGAHFLARSLDDALKLTERPELANKVDMIWIVGGSS

DHFR          IPEKNRPLKGRINLVLSRELKEPPQGAHFLSRSLDDALKLTEQPELANKVDMVWIVGGSS

Peptide       -----------INLVLSRE-----------------------------------------

                         ********

Peptide 78-91

DHFR2         IPEKNRPLKDRINLVLSRELKEPPQGAHFLARSLDDALKLTERPELANKVDMIWIVGGSS

DHFR          IPEKNRPLKGRINLVLSRELKEPPQGAHFLSRSLDDALKLTEQPELANKVDMVWIVGGSS

peptide      ------------------ELKEPPQGAHFLSRS---------------------------

                                ************:**

Peptide 138-1555

DHFR2         VYKEAMNHLGHLKLFVTRIMQDFESDTFFSEIDLEKYKLLPEYPGVLSDVQEGKHIKYKF

DHFR          VYKEAMNHPGHLKLFVTRIMQDFESDTFFPEIDLEKYKLLPEYPGVLSDVQEEKGIKYKF

peptide      -----------------RIMQDFESDTFFPEIDLEKY-----------------------

                               ************ *******

Peptide 158-173

DHFR2         VYKEAMNHLGHLKLFVTRIMQDFESDTFFSEIDLEKYKLLPEYPGVLSDVQEGKHIKYKF

DHFR          VYKEAMNHPGHLKLFVTRIMQDFESDTFFPEIDLEKYKLLPEYPGVLSDVQEEKGIKYKF

peptide      -------------------------------------KLLPEYPGVLSDVQEEKG-----

                                                   *************** *

Peptide 158-176

DHFR2         VYKEAMNHLGHLKLFVTRIMQDFESDTFFSEIDLEKYKLLPEYPGVLSDVQEGKHIKYKF

DHFR          VYKEAMNHPGHLKLFVTRIMQDFESDTFFPEIDLEKYKLLPEYPGVLSDVQEEKGIKYKF

peptide      -------------------------------------KLLPEYPGVLSDVQEEKGIKY--

                                                    *************** * ***

**Figure S3 Identification of DHFR peptides from Band 1 of HepG2 mitochondria gel purified 21kDa extraction and subsequent amino acid sequence alignment of identified peptides with DHFR and DHFR2**


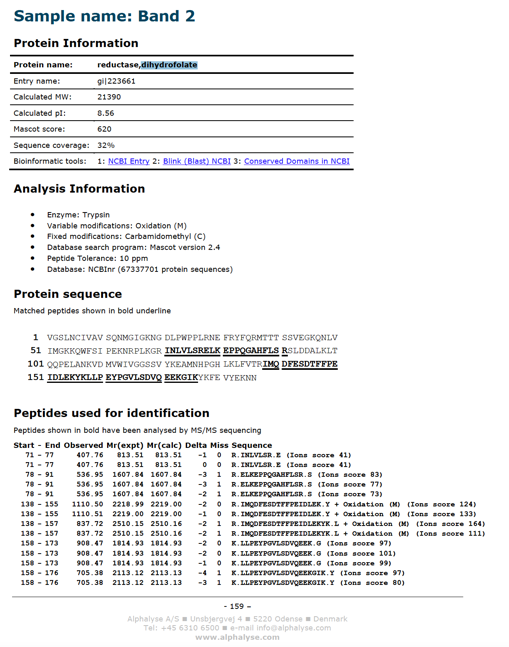


**Band 2**

Peptide 71-77

DHFR2         IPEKNRPLKDRINLVLSRELKEPPQGAHFLARSLDDALKLTERPELANKVDMIWIVGGSS

DHFR          IPEKNRPLKGRINLVLSRELKEPPQGAHFLSRSLDDALKLTEQPELANKVDMVWIVGGSS

peptide       ----------RINLVLSRE-----------------------------------------

                        *********

Peptide 78-91

DHFR2         IPEKNRPLKDRINLVLSRELKEPPQGAHFLARSLDDALKLTERPELANKVDMIWIVGGSS

DHFR          IPEKNRPLKGRINLVLSRELKEPPQGAHFLSRSLDDALKLTEQPELANKVDMVWIVGGSS

peptide       -----------------RELKEPPQGAHFLSRS---------------------------

                               *************:**

Peptide 138-155

DHFR2         VYKEAMNHLGHLKLFVTRIMQDFESDTFFSEIDLEKYKLLPEYPGVLSDVQEGKHIKYKF

DHFR          VYKEAMNHPGHLKLFVTRIMQDFESDTFFPEIDLEKYKLLPEYPGVLSDVQEEKGIKYKF

pepitide      -----------------RIMQDFESDTFFPEIDLEKY-----------------------

                               ************ *******

Peptide 138-157

DHFR2         VYKEAMNHLGHLKLFVTRIMQDFESDTFFSEIDLEKYKLLPEYPGVLSDVQEGKHIKYKF

DHFR          VYKEAMNHPGHLKLFVTRIMQDFESDTFFPEIDLEKYKLLPEYPGVLSDVQEEKGIKYKF

pepitide      -----------------RIMQDFESDTFFPEIDLEKYKL---------------------

                               ************ *********

Peptide 158-173

DHFR2         VYKEAMNHLGHLKLFVTRIMQDFESDTFFSEIDLEKYKLLPEYPGVLSDVQEGKHIKYKF

DHFR          VYKEAMNHPGHLKLFVTRIMQDFESDTFFPEIDLEKYKLLPEYPGVLSDVQEEKGIKYKF

peptide       -------------------------------------KLLPEYPGVLSDVQEEKG-----

                                                   *************** *

Peptide 158-176

DHFR2         VYKEAMNHLGHLKLFVTRIMQDFESDTFFSEIDLEKYKLLPEYPGVLSDVQEGKHIKYKF

DHFR          VYKEAMNHPGHLKLFVTRIMQDFESDTFFPEIDLEKYKLLPEYPGVLSDVQEEKGIKYKF

peptide       -------------------------------------KLLPEYPGVLSDVQEEKGIKY--

                                                   *************** * ***

**Figure S4 Identification of DHFR peptides from Band 2 of HepG2 mitochondria gel purified 21kDa and subsequent amino acid sequence alignment of identified peptides with DHFR and DHFR2.**


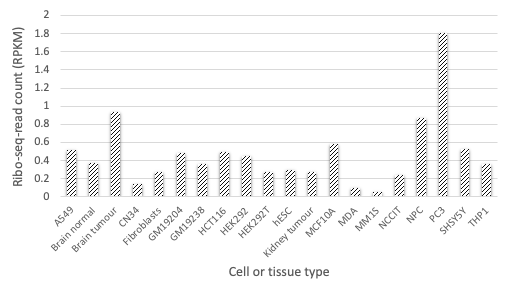


**Figure S5. Bioinformatic analysis of published Ribo-seq datasets from the RPFdb v2.0 beta database reveals that the DHFR2 RNA binds to the ribosome.** The RPKM values for the *DHFR2* gene was extracted from the 20 quality-controlled samples and assessed to determine whether its RNA associates with the ribosome. The RPKM values are the normalised unit that measures the level at which an RNA molecule binds to the ribosome. This data shows that the ribosome associates with the DHFR2 RNA.

**Table S3. Dihydrofolate reductase peptides detected in the DDA LC-MS/MS analysis on the panel of human cell lines and tissues**

| **Peptide Sequence** | **Modifications** | **Protein Name** | **HepG2** | **iPS** | **HuH7** | **IMR32** | **NEP** | **Testis** |
| --- | --- | --- | --- | --- | --- | --- | --- | --- |
| MTTTSSVEGK | - | DHFR / DHFR2 | x | ✓ | ✓ | x | ✓ | x |
| INLVLSR | - | DHFR / DHFR2 | ✓ | x | x | x | x | x |
| NGDLPWPPLR | - | DHFR | x | x | x | ✓ | x | x |
| LTEQPELANK | - | DHFR | x | ✓ | ✓ | x | x | ✓ |
| VDMVWIVGGSSVYK | - | DHFR | x | ✓ | x | ✓ | x | x |
| EAmNHPGHLK | Oxidation (M) | DHFR | ✓ | x | x | x | x | x |
| EAMNHPGHLK | - | DHFR | ✓ | ✓ | x | x | x | x |
| IMQDFESDTFFPEIDLEK | - | DHFR | ✓ | x | x | x | x | x |
| LLPEYPGVLSDVQEEK | - | DHFR | ✓ | ✓ | x | ✓ | x | x |
| YKFEVYEK | - | DHFR | x | ✓ | x | x | ✓ | x |

**
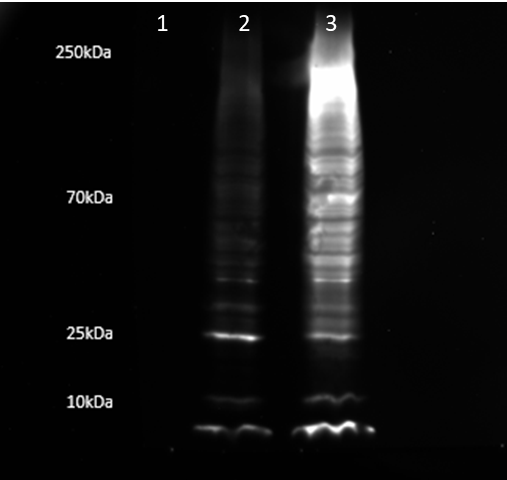
**

**Figure S6. Proteasome inhibitor MG132 blocks proteolytic activity of the 26S proteasome complex halting the degradation of ubiquitinated proteins**. An immunoblot was performed to confirm the HepG2 cells treated with MG132 resulted in a build-up of ubiquitinated proteins compared to the DMSO control. Lane 1, 10 – 250 kDa protein ladder; Lane 2, DMSO treated control with low levels of ubiquitinated proteins; Lane 3, MG132 treated cells with high levels of ubiquitinated proteins.

**Table S4. Dihydrofolate reductase peptides detected in the DDA LC-MS/MS analysis on DMSO and MG132 treated HepG2 cells**

| **Peptide Sequence** | **Modifications** | **Protein Name** | **DMSO Control Sample** | **MG132 Sample** |
| --- | --- | --- | --- | --- |
| MTTTSSVEGK | - | DHFR/DHFR2 | ✓ | ✓ |
| QNLVIMGK | - | DHFR | ✓ | ✓ |
| EPPQGAHFLSR | - | DHFR | ✓ | ✓ |
| LTEQPELANK | - | DHFR | ✓ | ✓ |
| VDMVWIVGGSSVYK | - | DHFR | ✓ | ✓ |
| IMQDFESDTFFPEIDLEK | - | DHFR | ✓ | X |
| LLPEYPGVLSDVQEEK | - | DHFR | ✓ | ✓ |

Table S5. Peptides identified in the Carnegie stage 21-22, post-conceptual week 9-10 and post-conceptual week 9-10 tissue

| **Protein name** | **Peptide sequence** | **Carnegie stage 21-22** | | | | | **Post-conceptual week 9-10** | | | | | **Post-conceptual week 15-17** | | | | |
| --- | --- | --- | --- | --- | --- | --- | --- | --- | --- | --- | --- | --- | --- | --- | --- | --- |
|  |  | **Kidney** | **Heart** | **Lung** | **Liver** | **Brain** | **Kidney** | **Heart** | **Lung** | **Liver** | **Brain** | **Kidney** | **Heart** | **Lung** | **Liver** | **Brain** |
| DHFR2 | NGDLPRPPLR | x | x | x | x | x | x | x | x | x | x | x | x | x | x | x |
|  | QNLVIMGR | x | x | x | x | x | x | x | x | x | x | x | x | x | x | x |
|  | VDMIWIVGGSSVYK | x | x | x | x | x | x | x | x | x | x | x | x | x | x | x |
|  | LLPEYPGVLSDVQEGK | x | ✓ | x | x | x | x | ✓ | x | x | x | x | ✓ | x | x | x |
| ACTB | AGFAGDDAPR | ✓ | ✓ | ✓ | ✓ | ✓ | ✓ | ✓ | ✓ | ✓ | ✓ | ✓ | ✓ | ✓ | ✓ | ✓ |
|  | DLTDYLMK | ✓ | ✓ | ✓ | x | ✓ | ✓ | ✓ | ✓ | x | ✓ | ✓ | ✓ | ✓ | x | ✓ |
| TUBA1A | DVNAAIATIK | ✓ | ✓ | ✓ | ✓ | ✓ | ✓ | ✓ | ✓ | ✓ | ✓ | ✓ | ✓ | ✓ | ✓ | ✓ |
| LMNB1 | LVEVDSGR | ✓ | ✓ | ✓ | ✓ | ✓ | ✓ | ✓ | ✓ | ✓ | ✓ | ✓ | ✓ | ✓ | ✓ | ✓ |
|  | AGGPTTPLSPTR | ✓ | ✓ | ✓ | ✓ | ✓ | ✓ | ✓ | ✓ | ✓ | ✓ | ✓ | ✓ | ✓ | ✓ | ✓ |
| HSPD1 | GIIDPTK | ✓ | ✓ | ✓ | ✓ | ✓ | ✓ | ✓ | ✓ | ✓ | ✓ | ✓ | ✓ | ✓ | ✓ | ✓ |
| COX4I1 | VNPIQGLASK | ✓ | ✓ | ✓ | ✓ | ✓ | ✓ | ✓ | ✓ | ✓ | ✓ | ✓ | ✓ | ✓ | ✓ | ✓ |
| TOM20 | LPTISQR | ✓ | ✓ | ✓ | ✓ | ✓ | x | ✓ | x | ✓ | x | ✓ | ✓ | x | ✓ | ✓ |

**Figure S7 The fragmentation mass spectrum for peptide LLPEYPGVLSDVQEGK of recombinant DHFR2 protein.** Peptide LLPEYPGVLSDVQEGK was detected by LC-MS/MS in protein lysate from HepG2 cells overexpressing DHFR2 recombinant protein with the retention time of 5.7 mins. The fragment ions of the peptide were y3, y11, y12, y14 and b6 as labelled in the spectrum and indicated in the peptide sequence shown. The predominant fragment ion was y14 as expected due to the position of the proline at y14 and possibly for the same reason, the next most abundant fragment ion of y11 also has a proline. Therefore, initially MRM transitions of y14 (871>759) and y11 (781>1128) were selected for identification of this peptide in human embryonic tissues. However, y11 transition was not found in the protein lysate of human embryonic tissue, this may be due to different inferencing ions present in the biological matrix of cell lines compared to human tissue. For this reason, y14 (871>759) and y12 (871>646) were used for the identification.
